# Impact of Remaining Teeth Number on Prognostic Nutritional Index in Patients Undergoing Gastrointestinal Tumor Surgery

**DOI:** 10.3390/nu18030514

**Published:** 2026-02-02

**Authors:** Mitsuyoshi Yoshida, Ryoko Igashira, Mieko Okamoto, Miyuki Yokoi, Yoshikazu Kobayashi, Nanako Hase-Kawata, Tsuyoshi Tanaka, Koichi Suda

**Affiliations:** 1 Department of Dentistry and Oral-Maxillofacial Surgery, School of Medicine, Fujita Health University, Toyoake 470-1192, Japan; fjnyd145@yahoo.co.jp (R.I.); mieko@fujita-hu.ac.jp (M.O.); miyuki.yokoi@fujita-hu.ac.jp (M.Y.); bread-t@fujita-hu.ac.jp (Y.K.); 2 Department of Dentistry and Oral-Maxillofacial Surgery, Fujita Health University Hospital, Toyoake 470-1192, Japan; nanako.kawata@fujita-hu.ac.jp; 3 Department of Surgery, School of Medicine, Fujita Health University, Toyoake 470-1192, Japan; tsuyoshi.tanaka@fujita-hu.ac.jp (T.T.); ko-suda@nifty.com (K.S.); 4 Collaborative Laboratory for Research and Development in Advanced Surgical Technology, Fujita Health University, Toyoake 470-1192, Japan

**Keywords:** number of teeth prognostic nutritional index, gastrointestinal cancers

## Abstract

**Objectives**: Malnutrition can influence perioperative complications and long-term survival in patients with gastrointestinal (GI) cancers. Oral functional decline is associated with decreased food intake, and a reduced number of remaining teeth contributes to malnutrition. However, the impact of preoperative oral conditions on perioperative nutritional status remains unclear. This study investigated the relationship between the number of remaining teeth and the prognostic nutritional index (PNI) in patients undergoing surgery for gastrointestinal malignancies. **Methods**: In total, 178 patients who underwent gastrointestinal surgery with perioperative oral management at our hospital between January and December 2022 were retrospectively analyzed. Data, including age, sex, tumor site, number of remaining teeth, blood test results, skeletal muscle index, and body mass index (BMI), were extracted from electronic medical records. The PNI and CRP–albumin ratio (CAR) were calculated. Correlations between the number of remaining teeth and nutritional indicators were examined. **Results**: The number of remaining teeth showed a significant correlation with the PNI in the upper gastrointestinal group (r = 0.336, *p* = 0.015) and with BMI in the hepatobiliary-pancreatic group (r = −0.519, *p* = 0.001), after adjusting for age using partial correlation analysis. No significant correlations were observed in the lower GI group. **Conclusions**: Among patients with upper GI cancer, a lower number of remaining teeth was associated with a lower PNI, influencing postoperative outcomes. Impaired oral function may affect the prognosis of patients with upper GI tumors, emphasizing the need for careful, comprehensive nutritional and oral management as part of perioperative support.

## 1. Introduction

Malnutrition in patients with gastric or colorectal cancer influences the development of postoperative complications, as well as short- and long-term survival outcomes [1,2]. According to the European Society for Clinical Nutrition and Metabolism guidelines, a decrease in food intake delays discharge by approximately 1 day, and the odds ratio for in-hospital death within 30 days increases with decreasing food intake [3]. Enhanced Recovery After Surgery (ERAS) programs have been introduced in recent years to prevent such complications and promote early postoperative recovery, with perioperative nutritional management being an important component. Furthermore, ERAS protocols for major surgery can reduce recovery time and hospital stay by 2–3 days and decrease complication rates by 30–50% [4].

In cancer patients, preoperative nutritional status and the degree of inflammation are associated with postoperative complications and prognosis; therefore, the prognostic nutritional index (PNI) was developed to evaluate these factors. The PNI can be calculated from routine blood tests—serum albumin levels and lymphocyte counts—and is considered a simple and useful assessment method [5].

Oral health is also related to the nutritional status of the elderly. Many previous studies have reported an association between nutritional intake and oral function [6]. A systematic review by Nitsuwat et al. [7] revealed that the number of remaining teeth and denture use are related to the intake of energy, protein, fat, and vitamin C. Moreover, individuals with <20 teeth tend to have a higher risk of malnutrition than those with ≥20 teeth [8]. Furthermore, it has been reported that individuals with fewer than 20 teeth are more prone to weight loss than those with 20 or more teeth [9]. This suggests that oral health status may even influence prognostic indicators for cancer patients.

In Japan, perioperative oral function management was integrated into the national health insurance system in 2012, establishing a framework for providing oral management services to patients undergoing general anesthesia. The necessity of perioperative oral management has also been demonstrated. Evidence has shown that improving oral hygiene preoperatively reduces the incidence of postoperative systemic complications [10,11]. However, although the importance of both perioperative nutritional management and the relationship between oral health and nutritional status has been recognized, the influence of preoperative oral condition on perioperative nutritional status remains unclear.

Therefore, in this study, we investigated the association between the preoperative PNI, which is often used as a predictive factor for complications and included in ERAS protocols, and the number of remaining teeth, one aspect of oral function, in patients undergoing surgery for GI tumors.

## 2. Materials and Methods

This study included patients who underwent surgery for GI tumors at the Department of Gastroenterological Surgery at our hospital between January and December 2022. This study was conducted with the approval of the Fujita Health University Review Committee (HM24-526). Patient details are described in the paper by Kawata et al. [12]. This study included only patients who underwent dental examinations.

The regular dental assessment evaluated tooth mobility, periodontal disease, the presence of caries, and the condition of prosthetic restorations. Teeth destroyed by caries were excluded, and dummy teeth with bridges were retained as remaining teeth. Necessary treatments such as tooth extraction and professional oral hygiene management were provided for these patients. Postoperative oral management continued, but this study focused solely on preoperative status.

Data extracted retrospectively from electronic medical records included age, sex, and tumor location. Patients were classified into three groups based on tumor location (the upper or lower GI or the hepato-biliary-pancreatic [HBP] region).

Blood test results were also extracted from the medical records. Serum albumin and CRP levels were measured using automated biochemical analyzers (LABOSPECT 008, Hitachi, Tokyo, Japan). White blood cell count and total lymphocyte count were calculated using an automated hematology analyzer (XN-3000, Sysmex Corporation, Tokyo, Japan).

The skeletal muscle mass index (SMI), body mass index (BMI), PNI, and *C*-reactive protein–albumin ratio (CAR) were calculated from laboratory findings as follows:

$$\begin{array}{c} \mathrm{BMI}=\mathrm{weight}(\mathrm{kg})/{\left(\mathrm{height}(m)\right)}^{2}\\ \mathrm{SMI}=\mathrm{appendicular}\;\mathrm{skeletal}\;\mathrm{muscle}\;\mathrm{mass}(\mathrm{kg})/{\left(\mathrm{height}(m)\right)}^{2}\\ \mathrm{PNI}=10\times \mathrm{Alb}\;(g/\mathrm{dL})+0.005\times \mathrm{TLC}\;(\mathrm{total}\;\mathrm{number}\;\mathrm{of}\;\mathrm{lymphocytes})\\ \mathrm{CAR}=\mathrm{CRP}\;(\mathrm{mg}/\mathrm{dL})/\mathrm{Alb}\;(g/\mathrm{dL}) \end{array}$$

The number of remaining teeth was used as the independent variable and examined for correlations with the SMI, BMI, PNI, and CAR. Partial correlation analysis was performed for variables showing significant correlations. Patients were divided into two groups: those with <20 teeth (considered at higher risk of malnutrition) and those with ≥20 teeth. The groups were compared using the Mann–Whitney U test and multiple regression analysis of significant variables. Statistical analyses were performed using IBM SPSS version 24.0 (IBM, Tokyo, Japan), with the significance level set at *p* < 0.05. Based on a previous study in past esophageal cancer patients showing a significant difference in the proportion of patients with two or fewer functional teeth in lower- and higher-PNI groups (54% vs. 20%) [13], the required sample size was calculated to be 75 cases.

## 3. Results

During the study period, 453 patients underwent surgery at the Department of Gastroenterological Surgery, of whom 178 received perioperative oral function management. Table 1 summarizes the patients’ characteristics. The upper GI group included 53 patients (41 with gastric cancer, 7 with esophageal cancer, and 5 with esophagogastric junction cancer), the HBP group included 84 patients (16 with pancreatic cancer, 16 with liver cancer, 5 with gallbladder cancer, and 4 with duodenal cancer), and the lower GI group included 41 patients (44 with colon cancer, 35 with rectal cancer, and 5 with cecal cancer). Furthermore, only a small number of patients received preoperative chemotherapy: four in the upper GI group, seven in the HBP group, and four in the lower GI group.

As shown in Table 2, after adjusting for age as a control variable, a significant partial correlation coefficient was indicated between the number of remaining teeth and the PNI in the upper GI group and the number of remaining teeth and BMI in the HBP group (*p* < 0.05).

Comparisons of the SMI, BMI, PNI, and CAR between groups based on the number of remaining teeth are shown in Table 3. In the upper GI group, the mean PNI value was 39.0 ± 4.4 in the ≥20-teeth group, which is significantly higher than the 35.1 ± 6.1 in the <20-teeth group (*p* < 0.05). The mean CAR was 0.2 ± 0.6 for the ≥20-teeth group, which was significantly lower than the 0.5 ± 1.2 for the <20-teeth group (*p* < 0.05). Furthermore, multiple regression analysis was conducted with the PNI as the dependent variable and the number of remaining teeth, age, and CAR as the independent variables. The results showed that the number of remaining teeth was significantly associated with the PNI independently (*p* = 0.026, 95% CI: 0.23–0.352).

In the HBP group, the mean BMI in the ≥20-teeth group was significantly lower than that in the <20-teeth group (*p* < 0.05). No significant differences were observed in the lower GI group.

## 4. Discussion

The results of this study indicate that patients with <20 teeth had significantly lower preoperative PNI values than those with ≥20 teeth, indicating that the former may be at higher risk of poor postoperative prognosis.

Interestingly, a significant decrease in the PNI was observed only in patients with upper GI disease, not in those with lower GI or HBP disease. Yamanaka-Kohno et al. [13] demonstrated a positive relationship between dental occlusal support and the PNI in patients with esophageal cancer who underwent esophagectomy. This study hypothesized postoperative dysphagia as a possible cause. Dysphagia, and certainly tooth loss itself, may contribute to low albumin levels by increasing the inflammatory response [14]. Postoperative dysphagia may be caused by inflammatory reactions; however, the causal relationship between the two factors remains unclear. We do believe that this may reflect the critical role of gastric digestion and nutrient absorption in postoperative outcomes, as functional disorders, such as delayed gastric emptying, dumping syndrome, or duodeno-gastro-esophageal reflux, occur in half of patients who undergo esophagectomy [15].

The mouth and stomach contribute significantly to the mechanical and, to some extent, chemical breakdown of starch, whereas the small intestine serves as a major site for nutrient absorption [16]. Sufficient breakdown of the physical structure of food is needed to ensure the efficiency of nutrient absorption from solid or semisolid food in the small intestine. According to Nadia et al. [17], if the ingested food that enters the stomach is a noncohesive mixture, it will need to undergo further macrostructural breakdown in the stomach. In such cases, the gastric emptying rate and glycemic response may be governed by the rate of food breakdown in the stomach and by gastric sieving.

The final particle size during oral processing is a key parameter in digestion. The main mastication (or chewing) agents are the teeth, which consist of different geometries that allow them to cut (the incisors), cut and tear (the canines and cuspids), or chew and shear (the molars) solid foods. Tooth loss reduces the ability to form food boluses [18]. Chewing is the process of repeatedly opening and closing the jaw and grinding food into small particles with the teeth for subsequent swallowing and digestion [19]. Chen et al. [20] demonstrated that the subjective masticatory ability of older adults can accurately indicate their masticatory performance. Furthermore, the quantity of teeth is a prevalent determinant of masticatory performance and ability. Loss of teeth, even if compensated for by removable dentures, hinders the formation of a normal bolus. Thus, the food boluses made by denture wearers contain many large-size particles [21]. For older adults, having ≥20 teeth is essential to maintaining optimal chewing function and a balanced nutritional intake [22]. However, no strong evidence has established the effect of tooth loss on diet and nutrition, with inconsistent results among the few studies that have investigated it [18]. When considering the relationship between tooth loss and nutritional status, compensatory functions through gastric digestion may also need to be considered. Patients with <20 remaining teeth and a low PNI likely experienced insufficient nutrient absorption due to reduced chewing capacity, leading to inadequate bolus formation. Conversely, no significant differences in the PNI were observed in patients with lower GI or HBP disease, likely because gastric function remains relatively intact and can compensate for reduced chewing. Given the critical impact of nutritional status on survival in gastric cancer, the findings indicate that oral care may play a key role in improving the nutritional status of patients with gastric cancer.

Furthermore, among patients with HBP disease, those with <20 remaining teeth had significantly higher BMI values, with a mean of 24.9, which is at the obesity threshold of 25. A large-scale study using health insurance data demonstrated an association between tooth loss and obesity among the general population [23,24]. Although this study could not determine whether the higher BMI reflected obesity or edema, previous reports indicate that reduced teeth quantity is associated with lower protein and fiber intake and higher carbohydrate intake [25], potentially contributing to sarcopenic obesity. A systematic review and meta-analysis conducted by Nascimento et al. [26] revealed a bi-directional association between tooth loss and obesity. Obesity is a known risk factor for HBP diseases, particularly pancreatitis, which itself is a risk factor for pancreatic cancer [27]. Ishikawa et al. [28] reported that lifestyle disorders leading to tooth loss contribute to weight gain. These results may indicate that maintaining good oral health helps reduce the risk of HBP disease through obesity prevention.

This study has several limitations. It has been noted that upper GI cancer patients are prone to malnutrition due to organic obstruction or cachexia [29]; however, this study did not evaluate gastric dysfunction itself. Additionally, upper GI surgery may cause pain during eating, chewing, and swallowing, and this issue is not thought to affect patients who undergo lower GI or HBP surgery to the same degree. Furthermore, psychological factors such as depression and anxiety may indirectly affect food intake. Oral function was assessed solely by the number of remaining teeth, without evaluating denture use, occlusal force, or masticatory performance. Data on dietary intake, food texture, and chewing difficulty were unavailable. As a single-center retrospective study, residual confounding cannot be excluded, and the findings may not be generalizable to other populations. Furthermore, whether prosthodontic intervention or occlusal rehabilitation can improve the PNI remains unknown and warrants future investigation. Despite these limitations, this study highlights the potential importance of oral function in perioperative nutritional management, particularly in patients undergoing upper GI surgery. Comprehensive oral assessment and targeted dental interventions may serve as valuable components of multidisciplinary perioperative care.

## 5. Conclusions

Among patients with upper GI cancer, a lower number of remaining teeth was associated with worse PNI, influencing postoperative outcomes. Impaired oral function may affect the prognosis of patients with upper GI tumors, emphasizing the need for careful, comprehensive nutritional and oral management as part of perioperative support.

## Figures and Tables

**Table 1:** Number of participants and mean values by surgical region.

		Upper GI	HBP	Lower GI
Number of participants (*n*)		53	41	84
Sex (*n*)	Male	40	29	47
	Female	13	12	37
Mean age (years)		75.8 ± 6.0	76.3 ± 4.8	75.8 ± 6.8
Number of remaining teeth		16.2 ± 9.1	17.3 ± 7.9	14.9 ± 8.9
SMI (kg/m^2^)		6.8 ± 0.8	7.3 ± 0.7	5.5 ± 1.7
BMI (kg/m^2^)		22.3 ± 3.8	23.1 ± 3.6	22.8 ± 4.2
WBC (/μL)		6.22 ± 2.1	5.5 ± 1.5	6.8 ± 3.4
TLC (/μL)		1371.7 ± 556.1	1486.4 ± 688.7	1517.2 ± 556.1
ALB (g/dL)		3.7 ± 0.6	3.6 ± 0.6	3.6 ± 0.5
CRP (mg/dL)		0.9 ± 2.4	0.6 ± 1.1	1.3 ± 3.9
PNI		37.2 ± 5.6	36.0 ± 6.1	35.7 ± 5.5
CAR		0.3 ± 0.9	0.2 ± 0.4	0.5 ± 1.8

GI: gastrointestinal; HBP: hepato-biliary-pancreatic; SMI: skeletal muscle mass index; BMI: body mass index; WBC: white blood cell; TLC: total lymphocyte count; ALB: serum albumin level; CRP: *C*-reactive protein; PNI: prognostic nutritional index; CAR: CRP–albumin ratio.

**Table 2:** Partial correlation coefficient between the number of remaining teeth and clinical parameters.

Control Variable	Variable	Group	Partial Correlation Coefficient	SMI	BMI	PNI	CAR
Age	Number of remaining teeth	Upper GI	r	−0.200	0.160	0.336	−0.134
			*p*-value	0.556	0.262	0.015 *	0.360
		HBP	r	−0.456	−0.519	0.023	0.029
			*p*-value	0.544	0.001 *	0.886	0.879
		Lower GI	r	0.601	−0.007	0.058	−0.090
			*p*-value	0.207	0.959	0.648	0.504

*: Partial correlation coefficient. GI: gastrointestinal; HBP: hepato-biliary-pancreatic; SMI: skeletal muscle mass index; BMI: body mass index; PNI: prognostic nutritional index; CAR: *C*-reactive protein–albumin ratio.

**Table 3:** Comparisons between surgical groups based on the number of remaining teeth.

A. Upper GI			
	≥20 Teeth	<20 Teeth	*p*-value
Number of participants (*n*)	28	25	
Mean age (years)	73.6 ± 5.8	78.2 ± 5.6	0.006 *
SMI	6.7 ± 0.5	7.1 ± 1.6	0.864
BMI	22.5 ± 3.1	22.0 ± 4.5	0509
PNI	39.0 ± 4.4	35.1 ± 6.1	0.012 *
CAR	0.2 ± 0.6	0.5 ± 1.2	0.043 *
B. HBP			
	≥20 Teeth	<20 Teeth	*p*-value
Number of participants (*n*)	23	18	
Mean age (years)	76.7 ± 5.1	75.9 ± 4.5	0.683
SMI	6.8 ± 0.6	7.6 ± 0.7	0.400
BMI	21.9 ± 2.8	24.9 ± 3.8	0.006 *
PNI	35.2 ± 6.2	37.0 ± 6.9	0.546
CAR	0.2 ± 0.4	0.2 ± 0.2	0.722
C. Lower GI			
	≥20 Teeth	<20 Teeth	*p*-value
Number of participants (*n*)	30	54	
Mean age (years)	74.2 ± 6.2	76.7 ± 7.0	0.140
SMI	6.1 ± 0.8	5.2 ± 2.0	0.527
BMI	22.8 ± 4.1	22.7 ± 4.3	0.842
PNI	36.0 ± 5.9	35.5 ± 5.3	0.661
CAR	0.3 ± 0.7	0.6 ± 2.1	0.492

*: *p* < 0.05, Mann–Whitney U test. GI: gastrointestinal; HBP: hepato-biliary-pancreatic; SMI: skeletal muscle mass index; BMI: body mass index; PNI: prognostic nutritional index; CAR: *C*-reactive protein–albumin ratio.

## Data Availability

The original contributions presented in this study are included in the article. Further inquiries can be directed to the corresponding author.

## Abbreviations

The following abbreviations are used in this manuscript:

BMI	Body mass index
CAR	CRP–albumin ratio
ERAS	Enhanced recovery after surgery
HBP	Hepato-biliary-pancreatic
PNI	Prognostic nutritional index
SMI	Skeletal muscle mass index

## References

[1] Ho J.W., Wu A.H., Lee M.W., Lau S.Y., Lam P.S., Lau W.S., Kwok S.S., Kwan R.Y., Lam C.F., Tam C.K. (2015). Malnutrition risk predicts surgical outcomes in patients undergoing gastrointestinal operations: Results of a prospective study. Clin. Nutr..

[2] Karanikki E., Frountzas M., Lidoriki I., Kozadinos A., Mylonakis A., Tsikrikou I., Kyriakidou M., Toutouza O., Koniaris E., Theodoropoulos G.E. (2024). The predictive role of preoperative malnutrition assessment in postoperative outcomes of patients undergoing surgery due to gastrointestinal cancer: A cross-sectional observational study. J. Clin. Med..

[3] Lobo D.N., Gianotti L., Adiamah A., Barazzoni R., Deutz N.E.P., Dhatariya K., Greenhaff P.L., Hiesmayr M., Jakobsen D.H., Klek S. (2020). Perioperative nutrition: Recommendations from the ESPEN expert group. Clin. Nutr..

[4] Ljungqvist O. (2014). ERAS--enhanced recovery after surgery: Moving evidence-based perioperative care to practice. JPEN J. Parenter. Enteral. Nutr..

[5] Lee J.Y., Kim H.I., Kim Y.N., Hong J.H., Alshomimi S., An J.Y., Cheong J.H., Hyung W.J., Noh S.H., Kim C.B. (2016). Clinical significance of the Prognostic Nutritional Index for predicting short- and long-term surgical outcomes after gastrectomy: A retrospective analysis of 7781 gastric cancer patients. Medicine.

[6] Yoshida M., Suzuki R., Kikutani T. (2014). Nutrition and oral status in elderly people. Jpn. Dent. Sci. Rev..

[7] Nitsuwat S., Webster J., Sarkar A., Cade J. (2025). The association of oral processing factors and nutrient intake in community-dwelling older adults: A systematic review and meta-analysis. Nutrients.

[8] Lee S., Sabbah W. (2018). Association between number of teeth, use of dentures and musculoskeletal frailty among older adults. Geriatr. Gerontol. Int..

[9] Shiota C., Kusama T., Takeuchi K., Kiuchi S., Osaka K. (2023). Oral Hypofunction and Risk of Weight Change among Independent Older Adults. Nutrients.

[10] Suzuki H., Matsuo K., Okamoto M., Nakata H., Sakamoto H., Fujita M. (2019). Preoperative periodontal treatment and its effects on postoperative infection in cardiac valve surgery. Clin. Exp. Dent. Res..

[11] Sakai H., Kurita H., Kondo E., Tanaka H., Shimane T., Hashidume M., Yamada S.-I. (2024). Dental and oral management in the perioperative period of surgery: A scoping review. Jpn. Dent. Sci. Rev..

[12] Kawata N., Yoshida M., Sakai A., Tanaka T., Inaguma G., Suda K., Ohuchi A. (2025). Relationship between the number of remaining teeth and postoperative delirium in patients after gastrointestinal surgery. Geriatr. Gerontol. Int..

[13] Yamanaka-Kohno R., Shirakawa Y., Inoue-Minakuchi M., Yokoi A., Muro M., Kosaki H., Tanabe S., Fujiwara T., Morita M. (2021). Association of dental occlusal support with the Prognostic Nutritional Index in patients with esophageal cancer who underwent esophagectomy. Esophagus.

[14] Yoshihara A., Iwasaki M., Ogawa H., Miyazaki H. (2013). Serum albumin levels and 10-year tooth loss in a 70-year-old population. J. Oral Rehabil..

[15] Ukegjini K., Vetter D., Fehr R., Dirr V., Gubler C., Gutschow C.A. (2021). Functional syndromes and symptom-orientated aftercare after esophagectomy. Langenbeck’s Arch. Surg..

[16] Kiela P.R., Ghishan F.K. (2016). Physiology of Intestinal Absorption and Secretion. Best Pract. Res. Clin. Gastroenterol..

[17] Nadia J., Bronlund J., Singh R.P., Singh H., Bornhorst G.M. (2021). Structural breakdown of starch-based foods during gastric digestion and its link to glycemic response: In vivo and in vitro considerations. Compr. Rev. Food Sci. Food Saf..

[18] Gaewkhiew P., Sabbah W., Bernabé E. (2017). Does tooth loss affect dietary intake and nutritional status? A systematic review of longitudinal studies. J. Dent..

[19] Layton D.M., Morgano S.M., Att W., Freilich M.A., Ferro K.J., Kelly J.A., Muller F., Nguyen C.T., Salinas T.J., Shah K.C. (2023). Glossary of prosthodontic terms 2023: Tenth edition. J. Prosthet. Dent..

[20] Chen F.I., Chen J.H., Jeng J.H., Akifusa S., Liu H.Y. (2025). Association and relevant factors between objective masticatory performance and subjective masticatory ability among community-dwelling older adults. J. Dent. Sci..

[21] Preoteasa E., Oncescu Moraru A.M., Meghea D., Murariu Magureanu C., Preoteasa C.T. (2022). Food Bolus Properties in Relation to Dentate and Prosthetic Status. Healthcare.

[22] Takehara S., Karawekpanyawong R., Okubo H., Tun T.Z., Ramadhani A., Chairunisa F., Tanaka A., Wright F.A.C., Ogawa H. (2023). Oral Health Promotion under the 8020 Campaign in Japan-A Systematic Review. Int. J. Environ. Res. Public Health.

[23] Hayashi M., Morino K., Harada K., Miyazawa I., Ishikawa M., Yasuda T., Iwakuma Y., Yamamoto K., Matsumoto M., Maegawa H. (2022). Real-world evidence of the impact of obesity on residual teeth in the Japanese population: A cross-sectional study. PLoS ONE.

[24] Kang J., Larvin H., Pavitt S., Wu J. (2024). Higher prevalence of tooth loss in people with abdominal obesity but normal weight: Findings from the United States and Scottish populations. Clin. Exp. Dent. Res..

[25] Nakamura M., Ojima T., Nagahata T., Kondo I., Ninomiya T., Yoshita K., Arai Y., Ohkubo T., Murakami K., Nishi N. (2019). Having few remaining teeth is associated with a low nutrient intake and low serum albumin levels in middle-aged and older Japanese individuals: Findings from the NIPPON DATA2010. Environ. Health Prev. Med..

[26] Nascimento G.G., Leite F.R., Conceição D.A., Ferrúa C.P., Singh A., Demarco F.F. (2016). Is there a relationship between obesity and tooth loss and edentulism? A systematic review and meta-analysis. Obes. Rev..

[27] Rawla P., Thandra K.C., Sunkara T. (2019). Pancreatic cancer and obesity: Epidemiology, mechanism, and preventive strategies. Clin. J. Gastroenterol..

[28] Ishikawa S., Konta T., Susa S., Ishizawa K., Makino N., Ueno Y., Okuyama N., Iino M. (2023). Association of health behaviors, dietary habits, and oral health with weight gain after 20 years of age in community-dwelling Japanese individuals aged 40 years and older: A cross-sectional study. Clin. Oral Investig..

[29] Xu R., Chen X.D., Ding Z. (2022). Perioperative nutrition management for gastric cancer. Nutrition.

